# A Prospective Study on Metabolic Risk Factors and Gallbladder Cancer in the Metabolic Syndrome and Cancer (Me-Can) Collaborative Study

**DOI:** 10.1371/journal.pone.0089368

**Published:** 2014-02-21

**Authors:** Wegene Borena, Michael Edlinger, Tone Bjørge, Christel Häggström, Björn Lindkvist, Gabriele Nagel, Anders Engeland, Tanja Stocks, Susanne Strohmaier, Jonas Manjer, Randi Selmer, Steinar Tretli, Hans Concin, Goran Hallmans, Håkan Jonsson, Pär Stattin, Hanno Ulmer

**Affiliations:** 1 Department of Medical Statistics, Informatics and Health Economics, Innsbruck Medical University, Innsbruck, Austria; 2 Department of Public Health and Primary Health Care, University of Bergen, Bergen, Norway; 3 Norwegian Institute of Public Health, Oslo/Bergen, Norway; 4 Department of Surgical and Perioperative Sciences, Urology and Andrology, Umeå University, Umeå, Sweden; 5 Department of Internal Medicine, Division of Gastroenterology and Hepatology, Sahlgrenska University Hospital, Gothenburg, Sweden; 6 Institute of Epidemiology and Medical Biometry, Ulm University, Ulm, Germany; 7 Institute of Preventive Medicine, Copenhagen University Hospital, Copenhagen, Denmark; 8 Department of Surgery, Malmö University Hospital, Lund University, Malmö, Sweden; 9 Cancer Registry of Norway, Institute of Population-based Cancer Research, Montebello, Oslo, Norway; 10 Agency for Preventive and Social Medicine, Bregenz, Austria; 11 Department of Public Health and Clinical Medicine, Nutritional Research, Umeå University, Umeå, Sweden; 12 Department of Radiation Sciences, Oncology, Umeå University, Umeå, Sweden; University of Campinas, Brazil

## Abstract

**Objective:**

To investigate the association between metabolic risk factors (individually and in combination) and risk of gallbladder cancer (GBC).

**Methods:**

The metabolic syndrome and cancer project (Me-Can) includes cohorts from Norway, Austria, and Sweden with data on 578,700 men and women. We used Cox proportional hazard regression models to calculate relative risks of GBC by body mass index (BMI), blood pressure, and plasma levels of glucose, cholesterol, and triglycerides as continuous standardised variables and their standardised sum of metabolic syndrome (MetS) z-score. The risk estimates were corrected for random error in measurements.

**Results:**

During an average follow-up of 12.0 years (SD = 7.8), 184 primary gallbladder cancers were diagnosed. Relative risk of gallbladder cancer per unit increment of z-score adjusted for age, smoking status and BMI (except for BMI itself) and stratified by birth year, sex and sub-cohorts, was for BMI 1.31 (95% confidence interval 1.11, 1.57) and blood glucose 1.76 (1.10, 2.85). Further analysis showed that the effect of BMI on GBC risk is larger among women in the premenopausal age group (1.84 (1.23, 2.78)) compared to those in the postmenopausal age group (1.29 (0.93, 1.79)). For the other metabolic factors no significant association was found (mid blood pressure 0.96 (0.71, 1.31), cholesterol 0.84 (0.66, 1.06) and serum triglycerides 1.16 (0.82, 1.64)). The relative risk per one unit increment of the MetS z-score was 1.37 (1.07, 1.73).

**Conclusion:**

This study showed that increasing BMI and impaired glucose metabolism pose a possible risk for gallbladder cancer. Beyond the individual factors, the results also showed that the metabolic syndrome as an entity presents a risk constellation for the occurrence of gallbladder cancer.

## Introduction

Primary gallbladder cancer (GBC) is the most common biliary tract tumour and the sixth most common cancer affecting the gastrointestinal tract [1], [2]. It is a disease typically characterised by late diagnosis and poor outcome with a five year survival of only about 32% [3]. Although the presence of gallstones is considered to be an important risk factor, several other unidentified factors may be important in the development of gallbladder carcinoma. About 10 to 25% of patients with this disease do not have associated cholelithiasis and only a small proportion (1 to 3%) of patients that do have gallstones actually develop cancer [4].

Metabolic syndrome (MetS) is a constellation of factors related to insulin resistance including obesity, impaired glucose tolerance, dyslipidaemia and hypertension with varying definitions [5]. It has consistently been associated with an increased risk of cardiovascular diseases and diabetes type 2 [6], [7], and recently with risk of cancer at some sites like colorectal, prostate and liver cancers [8]–[13]. There is little data on the association between the MetS and risk of GBC, for separate as well as for a combination of MetS factors [10]–[17]. Most of these studies are either based on a single specific metabolic factor like obesity or diabetes [10]–[12], [14], apply an unfavourable proxy for MetS or they are non-prospective in nature [13]–[17]. To our knowledge this is the largest prospective study that assessed MetS and separate metabolic risk factors like serum lipids and blood pressure in association with gallbladder carcinoma.

In this large study of 578,700 participants, we aimed to investigate the association between metabolic risk factors, individually and in combination, and the risk of gallbladder cancer, taking random error into account.

## Materials and Methods

Detailed description of materials and methods of this study has been presented previously [18], [19].

### Study Population and Measurements

The study population comes from the Metabolic syndrome and Cancer project (Me-Can) which includes cohorts with 578,700 participants from Norway, Austria and Sweden. In these cohorts, health examinations data have been collected on height, weight, blood pressure, blood levels of glucose, total cholesterol, triglycerides, and smoking status. Time period of data collection spanned from 1972 to 2006. A detailed description of Me-Can and inclusion criteria for participants in this study has been previously described [18].

### Follow-up and Endpoints

Linkages have been performed with cause of death and vital status registries of the respective countries in order to identify those cases with incident gallbladder cancer (ICD-7): 155.1). Endpoints for the study were set at the date of the first cancer diagnosis, emigration, death, or December 31, 2003 (Austria), 2005 (Norway) and 2006 (Sweden).

### Statistical Analysis

The statistical analysis of this study is similar to a previously published study by the same study group [19]. In brief, Cox proportional hazards regression models, with age as the time variable, were fitted to obtain hazard ratios, denoted as relative risks (RRs), of primary GBC incidence with 95% confidence intervals (95% CI). We did our main analyses with both sexes combined as there was no significant interaction between sex and each of the MetS factors. As in the previous publications of Me-Can studies, analyses were undertaken with exposures as quintiles, standardized z-score continuous variables as well as bi-categorical values using the WHO defined cut-off points of the determinant variables.

### Quintile Analysis

Quintile cut-off points for the exposure variables were calculated within each cohort and sex. For glucose, cholesterol and triglycerides, cut-offs were additionally stratified by fasting time before blood sampling (>8 hours, fasting or ≤8 hours, non-fasting). The models were further stratified for the seven cohorts, sex and year of birth (five categories: ≤1929, 1930–39, 1940–49, 1950–59, and ≥1960), and adjusted for age, smoking status (three categories: never, former and current smokers) and for BMI where appropriate. The lowest quintile was used as a reference. Mean levels within the quintiles of exposure variables were used to test for linear trend.

### Standardized z-score Analysis

In addition to the quintile analysis, we also performed statistical tests with the exposures on a continuous scale. Standardized scores let each determinant to be investigated in the same scale making a uniform comparison possible. We transformed the existing values to standardised variables (z-scores), with zero as mean and one as standard deviation (z = (x - μ)/σ). As in the quintile analysis, the transformation is stratified by cohort, sex, and fasting time. Skewed variables (glucose and triglycerides) were logarithmically transformed prior to standardisation. Metabolic syndrome (MetS) score was constructed by adding the individual z-scores, and further standardization of the resulting sum. The adjustments and the stratifications in the z-score analysis are the same as in the quintile analysis.

### Analysis by WHO Standards

We also estimated risks in two categories according to cut-offs defined by WHO as follows: overweight (BMI 25–<30 kg/m^2^), obesity (BMI ≥30 kg/m^2^), hypertension (systolic blood pressure ≥140 mmHg and/or diastolic blood pressure ≥90 mmHg), impaired glucose tolerance (fasting glucose 6.0–6.9 mmol/l), diabetes (fasting glucose ≥7.0 mmol/l), hypertriglyceridemia (fasting triglycerides ≥1.7 mmol/l), and hypercholesterolemia (fasting total cholesterol ≥6.2 mmol/l). For blood glucose and lipids only those individuals who had fasted >8 hours prior to blood draw were included [20]–[22]. The same adjustment and stratification scheme was used as in the models with quintile and continuous exposure variables.

### Random Errors

All risk estimates were adjusted for random error in exposure measurements, based on data on repeated measurements from 133,820 participants with a total of 406,364 observations. These data were used to estimate regression dilution ratios (RDR) or regression calibration (RC) based on linear mixed effect models [23]–[25]. RRs derived from quintile and standardised z-score analyses were then corrected by dividing the regression coefficient in the Cox model by the estimated regression dilution ratio (RDR) of exposure. RRs from the z-scores analyses which adjusted for all individual metabolic factors in one model were corrected by regression calibration.

### Further Analytic Considerations

Since reproductive factors are important risk factor for gallbladder diseases in women [3], [4], we did additional risk estimation separately for women <50 years of age (n = 214,572) and ≥50 years of age (n = 72,748) using this age-cut-off as a proxy for pre- and postmenopausal status, respectively.

Our main analyses excluded the first year after baseline measurements in order to account for possible reverse causality between exposures and event. We consolidated the issue by performing further lag-time analyses that excluded the first 3 years of follow-up.

Statistical analyses were performed in Stata (version 10.0, StataCorp LP, College Station, Texas) and R (version 2.7.2, used for random error correction).

### Ethics

The study was approved by The Research Review Board of Umeå, Sweden, the Regional Committee for Medical and Health Research Ethics, Southeast Norway and the Ethikkommission of the Land Vorarlberg, Austria. Participants from Sweden and Austria provided written informed consent to participate in this study. In Norway, the participants were invited to come to the health survey and a questionnaire was sent together with the invitation. An attendance to the health examination where the participants delivered their filled in questionnaire, has been accepted by the Data Inspectorate as an informed consent, but not a written consent. Written consent was obtained from 1994 onwards.

## Results

Mean age at baseline was 43.9 years (SD = 11.1) in men and 44.1 years (SD = 12.3) in women (Table 1). Men were followed on average for 12.8 years (SD = 8.6) and women for 11.3 years (SD = 6.8). The prevalence of overweight or obesity (BMI 25 kg/m^2^ or higher) was 55% in men and 41% in women. Among participants with a follow-up time longer than one year 91 men and 93 women were diagnosed with primary GBC. Mean ages at the time of cancer diagnosis were 62.9 years (SD = 8.7) in men and 65.5 years (SD = 10.9) in women.

**Table 1:** Baseline characteristics of study participants in the Metabolic syndrome and Cancer project (Me-Can) including the first year of follow-up (n = 578,700).

Cohort(year of baseline measurement),n participants (%)	Men	Women
Oslo (1972–73)	16,760 (6)	
NCS (1974–83)	25,952 (9)	25,072 (9)
CONOR (1995–2003)	52,181 (18)	57,687 (20)
40-y (1994–99)	60,676 (21)	68,211 (23)
VHM&PP (1988–2002)	73,213 (25)	86,671 (30)
VIP (1985–2005)	38,843 (13)	40,669 (14)
MPP (1974–92)	22,241 (8)	10,524 (4)
Total (1972–2005)	289,866	288,834
Baseline age, years		
Mean (SD)	43.9 (11.1)	44.1 (12.3)
Categories, n (%)		
<30	27,244 (9)	33,067 (11)
30– <45	157,145 (54)	154,462 (54)
45– <60	76,623 (27)	67,689 (23)
60-	28,854 (10)	33,616 (12)
Fasting time, hours, n (%)1		
<4	120,510 (41)	122,319 (42)
4–8	30,769 (11)	26,802 (9)
>8	138,587 (48)	139,713 (49)
Smoking status, n (%)		
Never smoker	113,496 (39)	144,815 (50)
Ex-smoker	86,086 (30)	72,600 (25)
Current smoker	89,419 (31)	70,721 (25)
Missing	865 (0)	698 (0)
BMI, kg/m2		
Mean (SD)	25.7 (3.5)	24.9 (4.4)
Categories, n (%)		
<25	131,167 (45)	170,535 (59)
25– <30	127,846 (44)	82,869 (29)
30–	30,853 (11)	35,430 (12)
Follow-up, years		
Mean (SD)	12.8 (8.6)	11.3 (6.8)
Categories, n (%)		
<5	36,755 (13)	35,451 (12)
5 – <15	178,968 (62)	199,151 (69)
15 – <25	24,971 (8)	29,751 (10)
25–	48,172 (17)	24,481 (9)

1 Proportion of participants with a fasting time >8 h: 5% in the Norwegian cohorts, 90% in the VIP, and 100% in the VHM&PP and MPP.

Abbreviations: Oslo = Oslo study I; NCS = Norwegian Counties Study; CONOR = Cohort of Norway; 40-y = Age 40-programme; VHM&PP = Vorarlberg Heath Monitoring and Prevention Programme; VIP = Västerbotten Intervention Project; MPP = Malmö Preventive Project; SD = standard deviation; BMI = body mass index.

In quintile analysis, BMI and blood glucose were significantly associated with increases in risk of GBC (Table 2). The relative risk for the highest versus lowest quintile in models, adjusted for age, smoking status and BMI (except for BMI itself), stratified by birth years, sex and cohorts, and corrected for RDR, was 1.94 (95% CI 1.08, 3.51) for BMI and 5.38 (1.11, 26.5) for blood glucose.

**Table 2:** Risk of Primary gallbladder cancer in relation to quintiles of metabolic factors (n = 575,390).

		Primary gallbladder cancer (n = 184)					
		Mean (SD)	n, cases	Model^2^		Model^3^	
Exposures	Quintile level^1^			RR	95%CI	RR	95%CI
BMI	1	20.7 (1.3)	20	1.00			
(kg/m2)	2	23.0 (0.8)	26	1.12	0.58, 2.19		
	3	24.7 (0.8)	38	1.49	0.80, 2.76		
	4	26.8 (0.9)	47	1.70	0.93, 3.09		
	5	31.3 (2.6)	53	1.94	1.08, 3.51		
	P _trend_			0.08			
Mean BP§	1	8.2 (4.9)	20	1.00		1.00	
(mmHg)	2	96.9 (2.4)	27	1.37	0.48, 4.01	1.27	0.44, 3.74
	3	102.7 (2.3)	41	2.11	0.77, 5.75	1.86	0.68, 5.04
	4	109.8 (2.9)	35	1.02	0.36, 2.86	0.82	0.29, 2.32
	5	124.5 (9.5)	60	1.81	0.68, 4.81	1.25	0.45, 3.45
	P _trend_			0.47		0.92	
Glucose	1	4.2(0.5)	31	1.00		1.00	
(mmol/l)	2	4.8 (0.3)	34	2.51	0.47, 13.5	2.32	0.43, 12.7
	3	5.1 (0.3)	28	1.18	0.20, 7.11	1.07	0.18, 6.33
	4	5.5 (0.4)	38	3.14	0.60, 16.5	2.64	0.49, 13.9
	5	6.8 (2.0)	53	7.52	1.56, 36.1	5.38	1.11, 26.5
	P _trend_			0.01		0.04	
Cholesterol	1	4.2 (0.5)	27	1.00		1.00	
(mmol/l)	2	5.0 (0.3)	37	1.14	0.53, 2.47	1.11	0.52, 2.38
	3	5.6 (0.3)	34	0.74	0.33, 1.61	0.70	0.32, 1.53
	4	6.2 (0.3)	40	0.71	0.35, 1.53	0.66	0.31, 1.42
	5	7.4 (0.8)	46	0.67	0.32, 1.46	0.62	0.29, 1.32
	P _trend_			0.14		0.08	
Triglycerides§§	1	0.7 (0.2)	22	1.00		1.00	
(mmol/l)	2	1.0 (0.2)	30	1.48	0.45, 4.88	1.38	0.38, 2.00
	3	1.3 (0.3)	34	1.40	0.44, 4.49	1.20	0.21, 1.16
	4	1.9 (0.4)	46	2.50	0.84, 7.61	1.94	0.40, 1.97
	5	3.1 (1.7)	48	2.06	0.67, 6.28	2.90	0.30, 1.53
	P _trend_			0.12		0.50	

1 Quintile levels grouped by cohort and sex and for glucose, cholesterol and triglycerides further by fasting time. RRs were estimated from Cox regression models with attained age as time scale after excluding the first year after baseline measurement.

2 RRs were adjusted for smoking status and age at baseline, stratified by cohort, sex and categories of birth year. RRs are corrected for regression dilution bias by use of the regression dilution ratio (RDR); conversion into uncorrected RR = exp (log (RR)*RDR). RDR: BMI, 0.90; mean blood pressure, 0.54; glucose, 0.28; cholesterol, 0.66; triglycerides, 0.51. Glucose and triglycerides were logarithmically transformed.

3 RR were further adjusted for quintiles levels of BMI (except in BMI analysis).

§ value missing for 1 case.

§§ value missing for 4 cases.

Abbreviations: RR, relative risk; SD, standard deviation; BMI, body mass index; Mid BP, mean blood pressure; RDR, regression dilution ratio.

In multivariable adjusted analyses of z-scores, significant associations were found for a unit z-score increment of BMI (1.31 (1.11, 1.57)) and blood glucose (1.76 (1.10, 2.85)). The relative risk per unit increment of the MetS z-score was 1.37 (1.07, 1.73). In a further analysis where all the metabolic risk factors were calibrated and adjusted for each other, the significant association persisted only for BMI. No statistically significant association with GBC was observed for blood pressure, cholesterol and triglycerides (Table 3).

**Table 3:** Relative risk (95% CI) of primary gallbladder cancer, by z-scores of metabolic factors, and of the MetS score (n = 575,390).

	Primary gallbladder cancer (n = 184)					
	Model^1^		Model^2^		Model^3^	
Exposures	RR	95%CI	RR	95%CI	RR	95%CI
BMI	1.31	1.11, 1.57			1.23	1.03, 1.46
Mean blood pressure	1.10	0.82, 1.46	0.96	0.71, 1.31	0.97	0.82, 1.15
Glucose^4^	1.97	1.38, 3.22	1.76	1.10, 2.85	1.58	0.98, 2.54
Cholesterol	0.87	0.69, 1.11	0.84	0.66, 1.06	0.84	0.64, 1.10
Triglycerides^4^	1.14	0.98, 1.88	1.16	0.82, 1.64	1.11	0.77, 1.61
MetS	1.37	1.07, 1.73				

1 Relative risks were estimated from Cox regression models after excluding the first year of follow-up after baseline measurement, with attained age as time scale, stratified by cohort, sex and categories of birth year, adjusted for baseline age and smoking status, and corrected for regression dilution bias by use of regression dilution ratio (RDR); conversion into uncorrected RR = exp (log (RR)*RDR). RDR: BMI, 0.90; mean blood pressure, 0.54; log(glucose), 0.28; cholesterol, 0.66; log(triglycerides), 0.51.

2 Additionally adjusted for BMI.

3 Further adjusted for all the individual z-scores (except in MetS score analysis). In addition, z-scores, derived from original values, were calibrated.

4 Glucose and triglycerides were logarithmically transformed.

Abbreviations: CI, confidence interval; MetS, metabolic syndrome; BMI, body mass index; RR, relative risk.

There were no statistically significant interactions when testing effect modification of metabolic factors on GBC risk. Notable were however varying associations of BMI with GBC by age. The relative risk per unit increment of BMI was 1.84 (1.23, 2.78) in premenopausal (n = 32) and 1.29 (0.93, 1.79) (n = 61) in postmenopausal (≥50 years of age) women.

In analyses of the exposures in dichotomised categories according to the WHO classification of risk factors (Table 4), increases in risk were found for individuals with overweight (BMI above versus below 25 kg/m^2^) and individuals with impaired glucose metabolism (fasting blood glucose above versus below 6.0 mmol/l) with a relative risk of 1.52 (1.12, 2.10) and 1.62 (1.00, 2.62), respectively. These analyses were restricted to 278,300 individuals with >8 h fasting time.

**Table 4:** Relative risk (95% CI) of primary gallbladder cancer by WHO categories of metabolic factors (n = 575,390).

Exposures		Relative risks (95% CI)					
		n, cases	Model^2^		Model^3^		
	Cut-off levels^1^		RR	95%CI	RR	95%CI	
BMI	<25	77	1.00				
(kg/m^2^)	≥25	107	1.52	1.12, 2.10			
Systolic BP	<140	108	1.00		1.00		
(mmHg)	≥140	76	1.04	0.75, 1.44	0.93	0.66, 1.29	
Diastolic BP	<90	123	1.00		1.00		
(mmHg)	≥90	61	1.00	0.73, 1.37	0.96	0.74, 1.25	
Fasting glucose^4^(mmol/l)	<6.0	101	1.00		1.00		
	≥6.0	23	1.80	1.12, 2.88	1.62	1.00, 2.62	
Fasting total cholesterol^4^	<6.2	78	1.00		1.00		
(mmol/l)	≥6.2	46	0.90	0.62, 1.32	0.89	0.61, 1.29	
Fasting triglycerides^4^	<1.7	80	1.00		1.00		
(mmol/l)	≥1.7	44	1.22	0.83, 1.80	1.09	0.73, 1.62	

1 Cut-off levels are according to WHO definition.

2 RRs were estimated from Cox regression models with attained age as time scale, adjusted for smoking status and age at baseline, stratified by cohort, categories of birth year, and sex.

3 RRs were further adjusted for BMI (except in BMI analysis).

4 RRs were estimated only for individuals who had fasted 8 or more hours before baseline blood sampling (n = 277,300).

Abbreviations: BMI, body mass index; BP, blood pressure; CI, confidence interval; RR, relative risk.

Supplementary tables S1–S4 show sub-analyses of risks for men and women separately. Sex-specific risk estimates were similar to the combined analyses with some exceptions. Notably, the magnitude of the observed association between BMI and GBC was stronger and statistically significant in women.

## Discussion

In this large cohort study comprising of 578,700 men and women, a composite metabolic syndrome score, based on BMI, blood pressure, and circulating concentrations of glucose, total cholesterol and triglycerides, was significantly associated with GBC risk. Further analysis of single metabolic risk factors revealed that BMI and glucose were significantly associated with increased risk of GBC.

### Strengths and Limitations

The main strengths of our study are the large number of participants and its prospective design. We used data from population-based surveys in three countries, with almost complete coverage of data for measured exposure factors. The large number of repeated measurements within the study population allowed us to adjust for random error in the individual MetS factors. We also used high quality national registers in Austria, Norway, and Sweden for the follow-up regarding cancer diagnoses [26]–[28].

An important limitation of the study is the lack of data on gallstone status - a well established risk factor for GBC [3], [4], [29]. Gallstone status is also highly linked to the presence of metabolic risk factors like obesity, diabetes and dyslipidemia [17], [30]. With this constellation we cannot exclude the possible mechanistic role of gallstones in the association between metabolic risk factors and GBC as has been elegantly presented in previous prospective studies on obesity and diabetes [31], [32] as well as case-control studies [15]–[16]. However, it is also evident that a considerable proportion of individuals with GBC show no sign of cholelithiasis [3], [4], signifying the presence of other factors that may play important role gallbladder carcinogenesis.

Another limitation of the study is the noticeably small number of events, despite the large number participants, which might have contributed to the large confidence intervals seen especially in the quintile analyses. Lack of information on the reproductive history [3] among women may also be a limitation. However, we did a further risk estimation for women <50 and ≥50 years of age as a proxy for pre- and postmenopausal age groups, respectively. Our study is also limited by lack of data on socioeconomic status as well as some other behavioural aspects like alcohol consumption and physical activity. Moreover we lack data on treatment history of the abnormal metabolic factors like hypertension and dyslipidemia which might, to some extent, have confounded our risk estimate. For the most recent definition of the MetS [5] we lack data on specific factors like waist circumference and high density lipoprotein cholesterol which we had to replace with BMI and total cholesterol respectively. Consequently we have presented our results based on a metabolic syndrome score which we used as a proxy for the syndrome [33].

### Comparisons with the Literature

The observed significant association between metabolic syndrome and GBC in this study with large number of participants and adequately long follow-up period strengthens reports by a previous case-control study based on over 600 biliary tract cancers which also found significant role of Mets on GBC [17]. However, such studies may be limited by the timing of exposure and outcome not being able to exclude reverse causality. This may probably be the case with the lack of association between waist circumference and risk of GBC. Such deficiencies are better dealt with prospective studies of long follow up period. Although several prospective studies exist on individual metabolic factors [31], [32], literatures on the association between MetS as an entity and GBC are scarce [13]. In a previous prospective study [13] MetS was defined as simultaneous exposure to antihypertensive, hypoglycaemic and hypolypemic treatments which is a rather rough approximation of MetS. Compared to ours this previous study did not find statistically significant association between MetS and GBC which is a rather questionable finding in the face of evident significant association with most of the individual components.

Although several independent mechanisms are depicted to underpin the association between obesity and cancer, the mechanisms that link obesity with gallbladder cancer risk are unclear [34]–[36]. Many studies have identified obesity as a risk to be more pronounced in women, and suggested a possible role of sex hormones (mainly oestrogen) in the pathogenesis of GBC [8], [13], [29], [36]–[41]. There are even indications that sex hormone receptors exist on the tumour tissue [42]. Our results, that increasing BMI poses greater risk mainly in younger women of premenopausal age group, might be supportive of this mechanism. This finding is supported by another cohort study in Norway [13].

In tumours that depend on oestrogen for their growth, like breast and endometrial cancers, obesity is shown to be of greater risk in postmenopausal women [43]–[46]. In our study, however, the risk for GBC was higher for younger women below the age of 50. This observation might be due to chance, as we did not find a significant interaction between age and BMI. However, if confirmed in further studies, the clinicopathological mechanisms may be entirely different for GBC.

Blood glucose levels were shown to be associated with incidence of cancer overall and in several specific sites like the colon, pancreas, liver, and endometrium in previous studies [11], [28], [47]–[48]. Studies reporting specifically a link between GBC and blood glucose levels are almost inexistent [28]. The association between glucose and cancer risk in our study remained after adjustment for major putative confounders like BMI, smoking and age, indicating a possible causal link. However, biological mechanisms in the association between blood glucose and cancer are poorly understood. A large case-control study by associated with high blood pressure, none of these studies identified blood pressure as risk for GBC.

A case-control study on serum lipids and biliary tract cancers including gallbladder cancer showed that compared to controls, cases had significantly higher mean levels of serum triglyceride (STG) [15]. However, our study, based on prospective data analyses as well as other similar cohort studies did not confirm this finding [48], [52]. In the study by Andreotti et al serum measurement took place shortly after cancer diagnosis. In this constellation one cannot rule out a possible reverse causation due to disease effect [53].

In conclusion, our study showed that increasing BMI and blood glucose levels are possible risk factors for GBC. Obesity was seen to pose a greater risk among women in the premenopausal age. Beyond the individual factors, the results of our study show that the metabolic syndrome as an entity presents a risk constellation for the occurrence of gallbladder cancer. Considering the rise in temporal trend of BMI and blood glucose levels [48], [54], we would anticipate that the incidence ofShebl et al indicated that although diabetes could be a risk factor for gallstone formation, the association between diabetes and GBC can be explained only partly by the positive association between diabetes and gallstones [14].

The inverse association we observed between total cholesterol and GBC in women may be largely due to preclinical effects of the cancer on total serum cholesterol [49]. A lag-time sub-analysis excluding 3 years of follow up after baseline measurement, rendered the association non-significant although the direction of association persisted. This was also shown in another recently published Me-Can study on total serum cholesterol and cancer [50].

Studies on the association between blood pressure and GBC incidence are scarce [48], [51]. Although it was shown that several cancer sites might be significantly GBC might also increase.

## Supporting Information

File S1 Table S1. Risk of primary gallbladder cancer (n = 91) in relation to quintiles of metabolic factors in men (n = 288,070). Table S2. Risk of primary gallbladder cancer (n = 91) in relation to quintiles of metabolic factors in women (n = 287,320). Table S3. Risk of primary gallbladder cancer (n = 184) by unit increment of z-scores of the metabolic factors and of the MetS score in men (n = 288,070) and in women (n = 287,320). Table S4. Risk of primary gallbladder cancer (n = 184) by WHO categories of metabolic factors in men (n = 288,070) and in women (n = 287,320).
(DOC)

## References

[1] Jemal A, Siegel R, Ward E, Murray T, Xu J, Thun MJ (2007) Cancer statistics. CA Cancer J Clin 57: 43–66. 17237035 10.3322/canjclin.57.1.43

[2] Levy AD, Murakata LA, Rohrmann CA Jr (2001) Gallbladder carcinoma: radiologic-pathologic correlation. Radiographics 21: 295–314. 11259693 10.1148/radiographics.21.2.g01mr16295

[3] Lazcano-Ponce EC, Miquel JF, Muñoz N, Herrero R, Ferrecio C, et al (2001) Epidemiology and molecular pathology of gallbladder cancer. CA Cancer J Clin 51: 349–364. 11760569 10.3322/canjclin.51.6.349

[4] Misra S, Chaturvedi A, Misra NC, Sharma ID (2003) Carcinoma of the gallbladder. Lancet Oncol 4: 167–176. 12623362 10.1016/s1470-2045(03)01021-0

[5] Eckel RH, Grundy SM, Zimmet PZ (2005) The metabolic syndrome. Lancet 365: 1415–1428. 15836891 10.1016/S0140-6736(05)66378-7

[6] Lakka HM, Laaksonen DE, Lakka TA, Niskanen LK, Kumpusalo E, et al (2002) The metabolic syndrome and total and cardiovascular disease mortality in middle-aged men. JAMA 288: 2709–2716. 12460094 10.1001/jama.288.21.2709

[7] Ninomiya JK, L’Italien G, Criqui MH, Whyte JL, Gamst A, Chen RS (2004) Association of the metabolic syndrome with history of myocardial infarction and stroke in the Third National Health and Nutrition Examination Survey. Circulation 109: 42–46. 14676144 10.1161/01.CIR.0000108926.04022.0C

[8] Calle EE, Rodriguez C, Walker-Thurmond K, Thun MJ (2003) Overweight, obesity, and mortality from cancer in a prospectively studied cohort of U.S. adults. N Engl J Med 348: 1625–1638. 12711737 10.1056/NEJMoa021423

[9] Bergstrom A, Pisani P, Tenet V, Wolk A, Adami H (2001) Overweight as an avoidable cause of cancer in Europe. Int J Cancer 91: 421–430. 11169969 10.1002/1097-0215(200002)9999:9999<::aid-ijc1053>3.0.co;2-t

[10] SH, Ohrr H, Sull JW, Yun JE, Ji M, Samet JM (2005) Fasting serum glucose level and cancer risk in Korean men and women. JAMA 293: 194–202. 15644546 10.1001/jama.293.2.194

[11] Jee SH, Yun JE, Park EJ, Cho ER, Park IS, Sull JW, et al (2008) Body mass index and cancer risk in Korean men and women. Int J Cancer 123: 1892–1896. 18651571 10.1002/ijc.23719

[12] Stocks T, Rapp K, Bjorge T, Manjer J, Ulmer H, et al (2009) Blood glucose and risk of incident and fatal cancer in the metabolic syndrome and cancer project (Me-Can): analysis of six prospective cohorts. PLoS Med 6: e1000201. 20027213 10.1371/journal.pmed.1000201 PMC2791167

[13] Russo A, Autelitano M, Bisanti L (2008) Metabolic syndrome and cancer risk. Eur J Cancer 44: 293–297. 18055193 10.1016/j.ejca.2007.11.005

[14] Engeland A, Tretli S, Austad G, Bjørge T (2005) Height and body mass index in relation to colorectal and gallbladder cancer in two million Norwegian men and women. Cancer Causes Control 16: 987–996. 16132807 10.1007/s10552-005-3638-3

[15] Shebl FM, Andreotti G, Rashid A, Gao YT, Yu K, et al (2010) Diabetes in relation to biliary tract cancer and stones: a population-based study in Shanghai, China. Br J Cancer 103: 115–119. 20517308 10.1038/sj.bjc.6605706 PMC2905288

[16] Andreotti G, Chen J, Gao YT, Rashid A, Chang SC, et al (2008) Serum lipid levels and the risk of biliary tract cancers and biliary stones: A population-based study in China. Int J Cancer 122: 2322–2329. 18076041 10.1002/ijc.23307 PMC2860727

[17] Shebl FM, Andreotti G, Meyer TE, Gao YT, Rashid A, et al (2011) Metabolic syndrome and insulin resistance in relation to biliary tract cancer and stone risks: a population-based study in Shanghai. China. Br J Cancer 105: 1424–9. 21915122 10.1038/bjc.2011.363 PMC3241543

[18] Stocks T, Borena W, Strohmaier S, Bjorge T, Manjer J, et al (2010) Cohort Profile: The Metabolic syndrome and Cancer project (Me-Can). Int J Epidemiol 39: 660–667. 19380371 10.1093/ije/dyp186 PMC2878454

[19] Borena W, Strohmaier S, Lukanova A, Bjørge T, Lindkvist B, et al (2012) Metabolic risk factors and primary liver cancer in a prospective study of 578,700 adults. Int J Cancer. 131(1): 193–200. 10.1002/ijc.26338 21805476

[20] Definition, diagnosis and classification of diabetes mellitus and its complications. Report of a WHO consultation. Part 1: Diagnosis and classification of diabetes mellitus. World Health Organisation, 1999, Geneva.

[21] Obesity: preventing and managing the global epidemic. Report of a WHO consultation. World Health Organ Tech Rep Ser 894: 1–253. 11234459

[22] Roth GA, Fihn SD, Mokdad AH, Aekplakorn W, Hasegawa T, Lim SS (2011) High total serum cholesterol, medication coverage and therapeutic control: an analysis of national health examination survey data from eight countries. Bull World Health Organ 1 89(2): 92–101. 10.2471/BLT.10.079947 PMC3040378 21346920

[23] Clarke R, Shipley M, Lewington S, Youngman L, Collins R, et al (1999) Underestimation of risk associations due to regression dilution in long-term follow-up of prospective studies. Am J Epidemiol 150: 341–353. 10453810 10.1093/oxfordjournals.aje.a010013

[24] Wood AM, White I, Thompson SG, Lewington S, Danesh J (2006) Regression dilution methods for meta-analysis: assessing long-term variability in plasma fibrinogen among 27,247 adults in 15 prospective studies. Int J Epidemiol 35: 1570–1578. 17148467 10.1093/ije/dyl233

[25] Fibrinogen Studies Collaboration (2009) Correcting for multivariate measurement error by regression calibration in meta-analyses of epidemiological studies. Stat Med 28: 1067–1092. 19222086 10.1002/sim.3530 PMC2930206

[26] Larsen IK, Smastuen M, Johannesen TB, Langmark F, Parkin DM, et al (2009) Data quality at the Cancer Registry of Norway: an overview of comparability, completeness, validity and timeliness. Eur J Cancer 45: 1218–1231. 19091545 10.1016/j.ejca.2008.10.037

[27] Barlow L, Westergren K, Holmberg L, Talback M (2009) The completeness of the Swedish Cancer Register: a sample survey for year 1998. Acta Oncol 48: 27–33. 18767000 10.1080/02841860802247664

[28] Rapp K, Schroeder J, Klenk J, Ulmer H, Concin H, et al (2006) Fasting blood glucose and cancer risk in a cohort of more than 140,000 adults in Austria. Diabetologia 49: 945–952. 16557372 10.1007/s00125-006-0207-6

[29] Hsing AW, Gao YT, Han TQ, Rashid A, Sakoda LC, et al (2007) Gallstones and the risk of biliary tract cancer: a population-based study in China. Br J Cancer 97: 1577–1582. 18000509 10.1038/sj.bjc.6604047 PMC2360257

[30] Méndez-Sánchez N, Chavez-Tapia NC, Motola-Kuba D, Sanchez-Lara K, Ponciano-Rodríguez G, et al (2005) Metabolic syndrome as a risk factor for gallstone disease. World J Gastroenterol. 11(11): 1653–7. 10.3748/wjg.v11.i11.1653 PMC4305948 15786544

[31] Schlesinger S, Aleksandrova K, Pischon T, Jenab M, Fedirko V, et al (2013) Diabetes mellitus, insulin treatment, diabetes duration, and risk of biliary tract cancer and hepatocellular carcinoma in a European cohort. Ann Oncol 24: 2449–55. 23720454 10.1093/annonc/mdt204 PMC3755330

[32] Schlesinger S, Aleksandrova K, Pischon T, Fedirko V, Jenab M, et al (2013) Abdominal obesity, weight gain during adulthood and risk of liver and biliary tract cancer in a European cohort. Int J Cancer 1 132: 645–57. 10.1002/ijc.27645 22618881

[33] Franks PW, Olsson T (2007) Metabolic syndrome and early death: getting to the heart of the problem. Hypertension 49(1): 10–2. 17130307 10.1161/01.HYP.0000251934.55488.ae

[34] Donohoe CL, Pidgeon GP, Lysaght J, Reynolds JV (2010) Obesity and gastrointestinal cancer. Br J Surg 97: 628–642. 20306531 10.1002/bjs.7079

[35] Roberts DL, Dive C, Renehan AG (2010) Biological mechanisms linking obesity and cancer risk: new perspectives. Annu Rev Med 61: 301–316. 19824817 10.1146/annurev.med.080708.082713

[36] Larsson SC, Wolk A (2007) Obesity and the risk of gallbladder cancer: a meta-analysis. Br J Cancer 96: 1457–1461. 17375043 10.1038/sj.bjc.6603703 PMC2360167

[37] Zatonski WA, Lowenfels AB, Boyle P, Maisonneuve P, Bueno de Mesquita HB, et al (1997) Epidemiologic aspects of gallbladder cancer: a case-control study of the SEARCH Program of the International Agency for Research on Cancer. J Natl Cancer Inst 89: 1132–1138. 9262251 10.1093/jnci/89.15.1132

[38] Moller H, Mellemgaard A, Lindvig K, Olsen JH (1994) Obesity and cancer risk: a Danish record-linkage study. Eur J Cancer 30: 344–350. 10.1016/0959-8049(94)90254-2 8204357

[39] Kuriyama S, Tsubono Y, Hozawa A, Shimazu T, Suzuki Y, et al (2005) Obesity and risk of cancer in Japan. Int J Cancer 113: 148–157. 15386435 10.1002/ijc.20529

[40] Renehan AG, Tyson M, Egger M, Heller RF, Zwahlen M (2008) Body-mass index and incidence of cancer: a systematic review and meta-analysis of prospective observational studies. Lancet 371: 569–578. 18280327 10.1016/S0140-6736(08)60269-X

[41] Renehan AG, Soerjomataram I, Tyson M, Egger M, Zwahlen M, et al (2010) Incident cancer burden attributable to excess body mass index in 30 European countries. Int J Cancer 126: 692–702. 19645011 10.1002/ijc.24803

[42] Percik R, Stumvoll M Obesity (2009) cancer (2009) Exp Clin Endocrinol Diabetes. 117: 563–6. 10.1055/s-0029-1241870 19924603

[43] Gupta P, Agarwal A, Gupta V, Singh PK, Pantola C, Amit S (2012) Expression and clinicopathological significance of estrogen and progesterone receptors in gallbladder cancer. Gastrointest Cancer Res 5: 41–47. 22690257 PMC3369597

[44] Cowey S, Hardy RW (2006) The metabolic syndrome: A high-risk state for cancer? Am J Pathol 169: 1505–1522. 17071576 10.2353/ajpath.2006.051090 PMC1780220

[45] Key TJ, Appleby PN, Reeves GK, Roddam A, Dorgan JF, et al (2003) Body mass index, serum sex hormones, and breast cancer risk in postmenopausal women. J Natl Cancer Inst 95: 1218–1226. 12928347 10.1093/jnci/djg022

[46] Kaaks R, Lukanova A, Kurzer MS (2002) Obesity, endogenous hormones, and endometrial cancer risk: a synthetic review. Cancer Epidemiol Biomarkers Prev. 11: 1531–1543. 12496040

[47] Stattin P, Björ O, Ferrari P, Lukanova A, Lenner P, et al (2007) Prospective study of hyperglycemia and cancer risk. Diabetes Care 30: 561–567. 17327321 10.2337/dc06-0922

[48] Tulinius H, Sigfússon N, Sigvaldason H, Bjarnadóttir K, Tryggvadóttir L (1997) Risk factors for malignant diseases: a cohort study on a population of 22,946 Icelanders. Cancer Epidemiol Biomarkers Prev 6: 863–873. 9367058

[49] Knekt P, Reunanen A, Aromaa A, Heliövaara M, Hakulinen T, et al (1988) Serum cholesterol and risk of cancer in a cohort of 39,000 men and women. J Clin Epidemiol 41: 519–530. 3290396 10.1016/0895-4356(88)90056-x

[50] Strohmaier S, Edlinger M, Manjer J, Stocks T, Bjørge T, et al (2013) Total serum cholesterol and cancer incidence in the Metabolic syndrome and Cancer Project (Me-Can). PLoS One. 8(1): e54242 doi:10.1371/journal.pone.0054242 10.1371/journal.pone.0054242 PMC3553083 23372693

[51] Stocks T, Van Hemelrijck M, Manjer J, Bjørge T, Ulmer H, et al (2012) Blood pressure and risk of cancer incidence and mortality in the Metabolic Syndrome and Cancer Project. Hypertension 59(4): 802–10. 22353615 10.1161/HYPERTENSIONAHA.111.189258

[52] Ulmer H, Borena W, Rapp K, Klenk J, Strasak A, et al (2009) Serum triglyceride concentrations and cancer risk in a large cohort study in Austria. Br J Cancer 6 101(7): 1202–6. 10.1038/sj.bjc.6605264 PMC2768093 19690552

[53] Iglesias A, Arranz M, Alvarez JJ, Perales J, Villar J, et al (1996) Cholesteryl ester transfer activity in liver disease and cholestasis, and its relation with fatty acid composition of lipoprotein lipids. Clin Chim Acta. 30 248(2): 157–74. 10.1016/0009-8981(95)06251-3 8740580

[54] Borena W, Stocks T, Strohmaier S, Strasak A, Manjer J, et al (2009) Long-term temporal trends in cardiovascular and metabolic risk factors. Wien Klin Wochenschr 121: 623–630. 19921129 10.1007/s00508-009-1238-z

