# Correction: A Prospective Study on Metabolic Risk Factors and Gallbladder Cancer in the Metabolic Syndrome and Cancer (Me-Can) Collaborative Study

**DOI:** 10.1371/journal.pone.0102291

**Published:** 2014-07-02

**Authors:** The PLOS ONE Staff

There are multiple errors in the article regarding the Funding statement, “Discussion” section and “Table 2.”

The correct Funding statement is:

“World Cancer Research Fund International for grant 2007/09 and Wereld Kanker Onderzoek Funds (WCRF NL) for grant R2010/247 to Pär Stattin and Medical University of Innsbruck (MUI START). The funders had no role in study design, data collection and analysis, decision to publish, or preparation of the manuscript.”

The correct order of the last five paragraphs of the “Discussion” section is:

"Blood glucose levels were shown to be associated with incidence of cancer overall and in several specific sites like the colon, pancreas, liver, and endometrium in previous studies [11, 28, 47-48]. Studies reporting specifically a link between GBC and blood glucose levels are almost inexistent [28]. The association between glucose and cancer risk in our study remained after adjustment for major putative confounders like BMI, smoking and age, indicating a possible causal link. However, biological mechanisms in the association between blood glucose and cancer are poorly understood. A large case-control study by Shebl et al indicated that although diabetes could be a risk factor for gallstone formation, the association between diabetes and GBC can be explained only partly by the positive association between diabetes and gallstones [14].

The inverse association we observed between total cholesterol and GBC in women may be largely due to preclinical effects of the cancer on total serum cholesterol [49]. A lag-time sub-analysis excluding 3 years of follow up after baseline measurement, rendered the association non-significant although the direction of association persisted. This was also shown in another recently published Me-Can study on total serum cholesterol and cancer [50].

Studies on the association between blood pressure and GBC incidence are scarce [48, 51]. Although it was shown that several cancer sites might be significantly associated with high blood pressure, none of these studies identified blood pressure as risk for GBC.

A case-control study on serum lipids and biliary tract cancers including gallbladder cancer showed that compared to controls, cases had significantly higher mean levels of serum triglyceride (STG) [15]. However, our study, based on prospective data analyses as well as other similar cohort studies did not confirm this finding [48, 52]. In the study by Andreotti et al serum measurement took place shortly after cancer diagnosis. In this constellation one cannot rule out a possible reverse causation due to disease effect [53].

In conclusion, our study showed that increasing BMI and blood glucose levels are possible risk factors for GBC. Obesity was seen to pose a greater risk among women in the premenopausal age. Beyond the individual factors, the results of our study show that the metabolic syndrome as an entity presents a risk constellation for the occurrence of gallbladder cancer. Considering the rise in temporal trend of BMI and blood glucose levels [48, 54], we would anticipate that the incidence of GBC might also increase."

The correct Table 2 can be found below:

**Table 2:** Risk of Primary gallbladder cancer in relation to quintiles of metabolic factors (n  =  575,390).

		Primary gallbladder cancer (n = 184)			
Exposures	Quintile level ^1^	Mean (SD)	n, cases	Model^2^RR 95%CI	Model^3^RR 95%CI
BMI	1	20.7 (1.3)	20	1.00	
(kg/m2)	2	23.0 (0.8)	26	1.12 0.58, 2.19	
	3	24.7 (0.8)	38	1.49 0.80, 2.76	
	4	26.8 (0.9)	47	1.70 0.93, 3.09	
	5	31.3 (2.6)	53	1.94 1.08, 3.51	
	P _trend_			0.08	
Mean BP^§^	1	8.2 (4.9)	20	1.00	1.00
(mmHg)	2	96.9 (2.4)	27	1.37 0.48, 4.01	1.27 0.44, 3.74
	3	102.7 (2.3)	41	2.11 0.77, 5.75	1.86 0.68, 5.04
	4	109.8 (2.9)	35	1.02 0.36, 2.86	0.82 0.29, 2.32
	5	124.5 (9.5)	60	1.81 0.68, 4.81	1.25 0.45, 3.45
	P _trend_			0.47	0.92
Glucose	1	4.2(0.5)	31	1.00	1.00
(mmol/l)	2	4.8 (0.3)	34	2.51 0.47, 13.5	2.32 0.43, 12.7
	3	5.1 (0.3)	28	1.18 0.20, 7.11	1.07 0.18, 6.33
	4	5.5 (0.4)	38	3.14 0.60, 16.5	2.64 0.49, 13.9
	5	6.8 (2.0)	53	7.52 1.56, 36.1	5.38 1.11, 26.5
	P _trend_			0.01	0.04
Cholesterol	1	4.2 (0.5)	27	1.00	1.00
(mmol/l)	2	5.0 (0.3)	37	1.14 0.53, 2.47	1.11 0.52, 2.38
	3	5.6 (0.3)	34	0.74 0.33, 1.61	0.70 0.32, 1.53
	4	6.2 (0.3)	40	0.71 0.35, 1.53	0.66 0.31, 1.42
	5	7.4 (0.8)	46	0.67 0.32, 1.46	0.62 0.29, 1.32
	P _trend_			0.14	0.08
Triglycerides^§§^	1	0.7 (0.2)	22	1.00	1.00
(mmol/l)	2	1.0 (0.2)	30	1.48 0.45, 4.88	1.38 0.38, 2.00
	3	1.3 (0.3)	34	1.40 0.44, 4.49	1.20 0.37, 3.84
	4	1.9 (0.4)	46	2.50 0.84, 7.61	1.94 0.64, 5.94
	5	3.1 (1.7)	48	2.06 0.67, 6.28	1.38 0.44, 4.34
	P _trend_			0.12	0.50

1 Quintile levels grouped by cohort and sex and for glucose, cholesterol and triglycerides further by fasting time. RRs were estimated from Cox regression models with attained age as time scale after excluding the first year after baseline measurement.

2 RRs were adjusted for smoking status and age at baseline, stratified by cohort, sex and categories of birth year. RRs are corrected for regression dilution bias by use of the regression dilution ratio (RDR); conversion into uncorrected RR  =  exp (log (RR)*RDR). RDR: BMI, 0.90; mean blood pressure, 0.54; glucose, 0.28; cholesterol, 0.66; triglycerides, 0.51. Glucose and triglycerides were logarithmically transformed.

3 RR were further adjusted for quintiles levels of BMI (except in BMI analysis).

§value missing for 1 case.

§§ value missing for 4 cases.

Abbreviations: RR, relative risk; SD, standard deviation; BMI, body mass index; Mid BP, mean blood pressure; RDR, regression dilution ratio
