# Supplementary material for: A Prospective Study on Metabolic Risk Factors and Gallbladder Cancer in the Metabolic Syndrome and Cancer (Me-Can) Collaborative Study
Source: PLoS One. 2014 Feb 21;9(2):e89368. doi: 10.1371/journal.pone.0089368 (PMC3931760; doi:10.1371/journal.pone.0089368)
Supplement: File S1 — Table S1. Risk of primary gallbladder cancer (n = 91) in relation to quintiles of metabolic factors in men (n = 288,070). Table S2. Risk of primary gallbladder cancer (n = 91) in relation to quintiles of metabolic factors in women (n = 287,320). Table S3. Risk of primary gallbladder cancer (n = 184) by unit increment of z-scores of the metabolic factors and of the MetS score in men (n = 288,070) and in women (n = 287,320). Table S4. Risk of primary gallbladder cancer (n = 184) by WHO categories of metabolic factors in men (n = 288,070) and in women (n = 287,320). (DOC) [file pone.0089368.s001.doc]

Table S1. Risk of primary gallbladder cancer (n=91) in relation to quintiles of metabolic factors in men (n= 288,070)

| Exposure | Quintile level | Mean (SD) | n | Model 1 ^1^ | | Model 2 ^2^ | |
| --- | --- | --- | --- | --- | --- | --- | --- |
|  |  |  |  | RR | 95% CI | RR | 95% CI |
|  |  |  |  |  |  |  |  |
| BMI | 1 | 21.5 (1.3) | 12 | 1.00 |  |  |  |
| (kg/m^2^) | 2 | 23.8 (0.8) | 15 | 1.15 | 0.49, 2.66 |  |  |
|  | 3 | 25.4 (0.8) | 21 | 1.51 | 0.68, 3.36 |  |  |
|  | 4 | 27.2 (0.9) | 21 | 1.42 | 0.64, 3.13 |  |  |
|  | 5 | 30.8 (2.6) | 22 | 1.50 | 0.68, 3.33 |  |  |
|  |  |  |  | P_trend_ = 0.27 | |  |  |
|  |  |  |  |  |  |  |  |
| Mid-BP ^3^ | 1 | 91.4 (4.9) | 11 | 1.00 |  | 1.00 |  |
| (mmHg) | 2 | 100.0 (2.4) | 19 | 2.16 | 0.52, 8.79 | 2.16 | 0.51, 8.47 |
|  | 3 | 105.6 (2.3) | 21 | 2.67 | 0.67, 10.7 | 2.49 | 0.62, 10.1 |
|  | 4 | 111.9 (2.9) | 13 | 0.75 | 0.16, 3.47 | 0.69 | 0.14, 3.21 |
|  | 5 | 125.6 (9.5) | 27 | 1.97 | 0.51, 7.76 | 1.69 | 0.42, 6.93 |
|  |  |  |  | P_trend_ = 0.86 | | P_trend_ = 0.93 | |
|  |  |  |  |  |  |  |  |
| Glucose | 1 | 4.2 (0.5) | 15 | 1.00 |  | 1.00 |  |
| (mmol/l) | 2 | 4.8 (0.3) | 16 | 3.81 | 0.32, 47.0 | 3.73 | 0.30, 45.5 |
|  | 3 | 5.2 (0.3) | 13 | 1.79 | 0.13, 24.7 | 1.69 | 0.13, 23.7 |
|  | 4 | 5.6 (0.4) | 20 | 7.06 | 0.60, 82.9 | 6.67 | 0.58, 78.5 |
|  | 5 | 7.0 (2.0) | 27 | 13.5 | 1.29, 139.9 | 12.3 | 1.14, 128.4 |
|  |  |  |  | P_trend_ = 0.02 | | P_trend_ = 0.03 | |
|  |  |  |  |  |  |  |  |
| Cholesterol | 1 | 4.3 (0.5) | 12 | 1.00 |  | 1.00 |  |
| (mmol/l) | 2 | 5.1 (0.3) | 18 | 1.33 | 0.42, 4.12 | 1.29 | 0.42, 4.05 |
|  | 3 | 5.7 (0.3) | 17 | 1.05 | 0.33, 3.31 | 1.02 | 0.32, 3.24 |
|  | 4 | 6.3 (0.3) | 21 | 1.24 | 0.41, 3.82 | 1.21 | 0.39, 3.67 |
|  | 5 | 7.4 (0.8) | 23 | 1.31 | 0.44, 3.94 | 1.24 | 0.42, 3.77 |
|  |  |  |  | P_trend_ = 0.70 | | P_trend_ = 0.78 | |
|  |  |  |  |  |  |  |  |
| Triglycerides ^4^ | 1 | 0.8 (0.2) | 15 | 1.00 |  | 1.00 |  |
| (mmol/l) | 2 | 1.2 (0.2) | 15 | 0.80 | 0.17, 3.75 | 0.78 | 0.16, 3.67 |
|  | 3 | 1.6 (0.3) | 9 | 0.24 | 0.04, 1.46 | 0.23 | 0.04, 1.41 |
|  | 4 | 2.1 (0.4) | 24 | 1.92 | 0.46, 7.81 | 1.80 | 0.42, 7.55 |
|  | 5 | 3.7 (1.7) | 25 | 2.11 | 0.52, 8.62 | 1.92 | 0.43, 8.35 |
|  |  |  |  | P_trend_ = 0.09 | | P_trend_ = 0.15 | |
|  |  |  |  |  |  |  |  |

^1^ RRs were estimated from Cox PH regression models with attained age as time scale after excluding the first year after baseline measurement; RRs are adjusted for smoking status and age at baseline, and stratified by cohort, sex and categories of birth year; RRs are corrected for regression dilution bias by use of the regression dilution ratio; conversion into uncorrected RR = exp (log(RR)*RDR); BMI RDR=0.90, mid-BP RDR=0.53, glucose RDR=0.30, cholesterol RDR=0.64, triglycerides RDR=0.46

^2^ Additionally adjusted for quintile levels of BMI (except for BMI)

^3^ Mid-BP is (systolic BP + diastolic BP) / 2

^4^ Value missing for 3 GBC cases

Abbreviations: BMI=body mass index; BP=blood pressure; GBC=gallbladder cancer; RDR=regression dilution ratio; RR=relative risk; SD=standard deviation

Table S2. Risk of primary gallbladder cancer (n=91) in relation to quintiles of metabolic factors in women (n=287,320)

| Exposure | Quintile level | Mean (SD) | n | Model 1 ^1^ | | Model 2 ^2^ | |
| --- | --- | --- | --- | --- | --- | --- | --- |
|  |  |  |  | RR | 95% CI | RR | 95% CI |
|  |  |  |  |  |  |  |  |
| BMI | 1 | 20.0 (1.2) | 8 | 1.00 |  |  |  |
| (kg/m^2^) | 2 | 22.3 (0.8) | 11 | 1.15 | 0.39, 3.35 |  |  |
|  | 3 | 24.1 (0.8) | 17 | 1.55 | 0.57, 4.20 |  |  |
|  | 4 | 26.4 (1.0) | 26 | 2.18 | 0.85, 5.64 |  |  |
|  | 5 | 31.7 (3.6) | 31 | 2.63 | 1.03, 6.72 |  |  |
|  |  |  |  | P_trend_ = 0.01 | |  |  |
|  |  |  |  |  |  |  |  |
| Mid-BP ^3^ | 1 | 85.0 (4.6) | 9 | 1.00 |  | 1.00 |  |
| (mmHg) | 2 | 93.6 (2.6) | 8 | 0.68 | 0.12, 3.84 | 0.62 | 0.11, 3.48 |
|  | 3 | 100.2 (3.0) | 20 | 1.67 | 0.39, 7.20 | 1.39 | 0.32, 6.03 |
|  | 4 | 107.7 (4.1) | 22 | 1.41 | 0.29, 5.29 | 0.89 | 0.20, 3.91 |
|  | 5 | 123.4 (11.2) | 33 | 1.67 | 0.39, 6.98 | 0.98 | 0.23, 4.31 |
|  |  |  |  | P_trend_ = 0.36 | | P_trend_ = 0.95 | |
|  |  |  |  |  |  |  |  |
| Glucose | 1 | 4.1 (0.5) | 16 | 1.00 |  | 1.00 |  |
| (mmol/l) | 2 | 4.7 (0.3) | 18 | 2.33 | 0.21, 26.6 | 2.08 | 0.18, 24.1 |
|  | 3 | 5.0 (0.3) | 15 | 0.18 | 0.01, 3.63 | 0.15 | 0.01, 3.04 |
|  | 4 | 5.4 (0.3) | 18 | 2.89 | 0.27, 31.7 | 2.26 | 0.21, 24.8 |
|  | 5 | 6.6 (1.7) | 26 | 3.37 | 0.33, 34.3 | 1.96 | 0.18, 20.5 |
|  |  |  |  | P_trend_ = 0.28 | | P_trend_ = 0.55 | |
|  |  |  |  |  |  |  |  |
| Cholesterol | 1 | 4.2 (0.4) | 15 | 1.00 |  | 1.00 |  |
| (mmol/l) | 2 | 4.9 (0.2) | 19 | 0.97 | 0.34, 2.79 | 0.94 | 0.33, 2.71 |
|  | 3 | 5.5 (0.3) | 17 | 0.48 | 0.16, 1.47 | 0.46 | 0.15, 1.39 |
|  | 4 | 6.1 (0.3) | 19 | 0.37 | 0.12, 1.11 | 0.35 | 0.16, 1.03 |
|  | 5 | 7.3 (0.9) | 23 | 0.27 | 0.12, 0.97 | 0.31 | 0.11, 0.90 |
|  |  |  |  | P_trend_ = 0.01 | | P_trend_ = 0.01 | |
|  |  |  |  |  |  |  |  |
| Triglycerides ^4^ | 1 | 0.7 (0.1) | 7 | 1.00 |  | 1.00 |  |
| (mmol/l) | 2 | 0.9 (0.1) | 15 | 3.61 | 0.50, 25.8 | 3.19 | 0.44, 23.0 |
|  | 3 | 1.1 (0.1) | 23 | 5.48 | 0.83, 35.8 | 4.35 | 0.67, 28.4 |
|  | 4 | 1.5 (0.2) | 24 | 4.90 | 0.76, 31.7 | 3.31 | 0.50, 21.8 |
|  | 5 | 2.5 (1.1) | 23 | 2.53 | 0.38, 17.1 | 1.33 | 0.18, 9.32 |
|  |  |  |  | P_trend_ = 0.59 | | P_trend_ = 0.72 | |
|  |  |  |  |  |  |  |  |

^1^ RRs were estimated from Cox PH regression models with attained age as time scale after excluding the first year after baseline measurement; RRs are adjusted for smoking status and age at baseline, and stratified by cohort, sex and categories of birth year; RRs are corrected for regression dilution bias by use of the regression dilution ratio; conversion into uncorrected RR = exp (log(RR)*RDR); BMI RDR=0.90, mid-BP RDR=0.56, glucose RDR=0.28, cholesterol RDR=0.66, triglycerides RDR=0.46

^2^ Additionally adjusted for quintile levels of BMI (except for BMI)

^3^ Mid-BP is (systolic BP + diastolic BP) / 2

^4^ Value missing for 1 GBC case

Abbreviations: BMI=body mass index; BP=blood pressure; GBC=gallbladder cancer; RDR=regression dilution ratio; RR=relative risk; SD=standard deviation

Table S3. Risk of primary gallbladder cancer (n=184) by unit increment of z-scores of the metabolic factors and of the MetS score in men (n= 288,070) and in women (n=287,320)

| Exposure | Men (n=91) | | | |  | Women (n=93) | | | |
| --- | --- | --- | --- | --- | --- | --- | --- | --- | --- |
|  | Model 1 ^1^ | | Model 2 ^2^ | |  | Model 1 ^1^ | | Model 2 ^2^ | |
|  | RR | 95% CI | RR | 95% CI |  | RR | 95% CI | RR | 95% CI |
|  |  |  |  |  |  |  |  |  |  |
| BMI | 1.17 | 0.91, 1.48 | 1.00 | 0.75, 1.35 |  | 1.51 | 1.17, 1.96 | 1.30 | 1.00, 1.71 |
|  |  |  |  |  |  |  |  |  |  |
| Mid-BP ^3^ | 0.91 | 0.59, 1.39 | 0.76 | 0.50, 1.17 |  | 1.05 | 0.67, 1.63 | 1.02 | 0.67, 1.56 |
|  |  |  |  |  |  |  |  |  |  |
| log(Glucose) | 2.26 | 1.07, 4.70 | 1.80 | 0.94, 3.42 |  | 1.42 | 0.68, 2.63 | 1.37 | 0.70, 2.70 |
|  |  |  |  |  |  |  |  |  |  |
| Cholesterol | 1.06 | 0.76, 1.48 | 1.07 | 0.75, 1.54 |  | 0.66 | 0.47, 0.93 | 0.65 | 0.44, 0.96 |
|  |  |  |  |  |  |  |  |  |  |
| log(Triglycerides) | 1.36 | 0.87, 2.15 | 1.25 | 0.74, 2.10 |  | 0.96 | 0.61, 1.51 | 1.02 | 0.60, 1.71 |
|  |  |  |  |  |  |  |  |  |  |
| MetS score | 1.41 | 1.00, 1.95 |  |  |  | 1.33 | 0.93, 1.90 |  |  |
|  |  |  |  |  |  |  |  |  |  |

^1^ RRs were estimated from Cox PH regression models with attained age as time scale after excluding the first year after baseline measurement; RRs are adjusted for smoking status and age at baseline, and stratified by cohort, sex and categories of birth year; RRs are corrected for regression dilution bias by use of the regression dilution ratio; conversion into uncorrected RR = exp (log(RR)*RDR); BMI RDR=0.90, mid-BP RDR=0.53, log(glucose) RDR=0.28, cholesterol RDR=0.64, log(triglycerides) RDR=0.51, MetS RDR=0.68 for men, and BMI RDR=0.90, mid-BP RDR=0.56, log(glucose) RDR=0.27, cholesterol RDR=0.66, log(triglycerides) RDR=0.50, MetS RDR=0.69 for women

^2^ Additionally adjusted for all the separate z-scores (except for MetS score); in addition, the z-scores were calibrated

^3^ Mid-BP is (systolic BP + diastolic BP) / 2

Abbreviations: BMI=body mass index; BP=blood pressure; CI=confidence interval; MetS=metabolic syndrome; RR=relative risk

Table S4. Risk of primary gallbladder cancer (n=184) by WHO categories of metabolic factors in men (n= 288,070) and in women (n=287,320).

| Exposure | Cut-off level ^1^ | Men (n=91) | | | | |  | Women (n=93) | | | | |
| --- | --- | --- | --- | --- | --- | --- | --- | --- | --- | --- | --- | --- |
|  |  | n | Model 1 ^2^ | | Model 2 ^3^ | |  | n | Model 1 ^2^ | | Model 2 ^3^ | |
|  |  |  | RR | 95% CI | RR | 95% CI |  |  | RR | 95% CI | RR | 95% CI |
|  |  |  |  |  |  |  |  |  |  |  |  |  |
| BMI | <25 | 39 | 1.00 |  |  |  |  | 38 | 1.00 |  |  |  |
| (kg/m^2^) | ≥25 | 52 | 1.28 | 0.84, 1.96 |  |  |  | 55 | 1.82 | 1.18, 2.82 |  |  |
|  |  |  |  |  |  |  |  |  |  |  |  |  |
| Systolic BP | <140 | 56 | 1.00 |  | 1.00 |  |  | 52 | 1.00 |  | 1.00 |  |
| (mmHg) | ≥140 | 35 | 1.09 | 0.70, 1.71 | 1.05 | 0.66, 1.65 |  | 41 | 0.98 | 0.61, 1.57 | 0.82 | 0.51, 1.34 |
|  |  |  |  |  |  |  |  |  |  |  |  |  |
| Diastolic BP | <90 | 62 | 1.00 |  | 1.00 |  |  | 61 | 1.00 |  | 1.00 |  |
| (mmHg) | ≥90 | 29 | 0.80 | 0.51, 1.26 | 0.76 | 0.48, 1.20 |  | 32 | 1.26 | 0.80, 1.97 | 1.06 | 0.67, 1.69 |
|  |  |  |  |  |  |  |  |  |  |  |  |  |
| Fasting glucose ^4^ | <6.0 | 51 | 1.00 |  | 1.00 |  |  | 50 | 1.00 |  | 1.00 |  |
| (mmol/l) | ≥6.0 | 9 | 1.37 | 0.66, 2.87 | 1.29 | 0.61, 2.74 |  | 14 | 2.22 | 1.20, 4.11 | 1.94 | 1.04, 3.64 |
|  |  |  |  |  |  |  |  |  |  |  |  |  |
| Fasting total cholesterol ^4^ | <6.2 | 37 | 1.00 |  | 1.00 |  |  | 41 | 1.00 |  | 1.00 |  |
| (mmol/l) | ≥6.2 | 23 | 1.23 | 0.72, 2.08 | 1.16 | 0.69, 1.94 |  | 23 | 0.68 | 0.40, 1.16 | 0.67 | 0.40, 1.13 |
|  |  |  |  |  |  |  |  |  |  |  |  |  |
| Fasting triglycerides ^4^ | <1.7 | 33 | 1.00 |  | 1.00 |  |  | 47 | 1.00 |  | 1.00 |  |
| (mmol/l) | ≥1.7 | 27 | 1.56 | 0.93, 2.61 | 1.45 | 0.87, 2.44 |  | 17 | 0.91 | 0.50, 1.66 | 0.77 | 0.42, 1.40 |
|  |  |  |  |  |  |  |  |  |  |  |  |  |

^1^ Cut-off levels are according to WHO definitions

^2^ RRs were estimated from Cox PH regression models with attained age as time scale after excluding the first year after baseline measurement; RRs are adjusted for smoking status and age at baseline, and stratified by cohort, sex and categories of birth year

^3^ Additionally adjusted for BMI (except for BMI)

^4^ RRs were estimated only for individuals who had fasted 8 hours or more before baseline blood sampling (n=138,587 in men and 139,713 in women)

Abbreviations: BMI=body mass index; BP=blood pressure; CI=confidence interval; RR=relative risk
